# An Early Reading Assessment Battery for Multilingual Learners in Malaysia

**DOI:** 10.3389/fpsyg.2020.01700

**Published:** 2020-07-10

**Authors:** Julia A. C. Lee, Seungjin Lee, Nur Fatihah Mat Yusoff, Puay Hoon Ong, Zaimuariffudin Shukri Nordin, Heather Winskel

**Affiliations:** ^1^Faculty of Cognitive Sciences and Human Development, Universiti Malaysia Sarawak, Kota Samarahan, Malaysia; ^2^Department of Education, Sehan University, Yeongam, South Korea; ^3^Dyslexia Association of Sarawak, Kuching, Malaysia; ^4^School of Health and Human Sciences, Southern Cross University, Lismore, NSW, Australia

**Keywords:** reading assessment battery, Malay language, transparent orthography, reading difficulties, reading disabilities, multilingual learners

## Abstract

The aim of the study was to develop a new comprehensive reading assessment battery for multi-ethnic and multilingual learners in Malaysia. Using this assessment battery, we examined the reliability, validity, and dimensionality of the factors associated with reading difficulties/disabilities in the Malay language, a highly transparent alphabetic orthography. In order to further evaluate the reading assessment battery, we compared results from the assessment battery with those obtained from the Malaysian national screening instrument. In the study, 866 Grade 1 children from multi-ethnic and multilingual backgrounds from 11 government primary schools participated. The reading assessment battery comprised 13 assessments, namely, reading comprehension, spelling, listening comprehension, letter name knowledge, letter name fluency, rapid automatized naming, word reading accuracy, word reading efficiency, oral reading fluency, expressive vocabulary, receptive vocabulary, elision, and phonological memory. High reliability and validity were found for the assessments. An exploratory factor analysis yielded three main constructs: phonological-decoding, sublexical-fluency, and vocabulary-memory. Phonological-decoding was found to be the most reliable construct that distinguished between at-risk and non-at-risk children. Identifying these underlying factors will be useful for detecting children at-risk for developing reading difficulties in the Malay language. In addition, these results highlight the importance of including a range of reading and reading-related measures for the early diagnosis of reading difficulties in this highly transparent orthography.

## Introduction

Becoming literate is an essential skill to be acquired in contemporary societies. Poor literacy skills can have multifaceted, devastating, and long-term consequences in relation to emotional, psychosocial, mental health, economic, and societal factors (Livingston et al., 2018). Specifically, children with reading difficulties are at risk for vicious cycles of struggles, failure, demoralization, lack of interest in school, emotional difficulties such as anxiety and frustration, behavioral problems such as aggressive-disruptive and delinquent-antisocial behaviors (Mugnaini et al., 2009), and psychosocial maladjustment such as interpersonal relationships and school adjustment (Greenham, 1999; Parhiala et al., 2014). Children with reading difficulties and learning disabilities tend to function poorly at school, which poses a risk factor for the onset of current and long-term psychological maladjustment (Mammarella et al., 2016). The reciprocal relation between anxiety and reading difficulties have been reported to have a predictive relation to lowered reading ability in the future (Livingston et al., 2018). Thus, the cycles of academic struggles lead to further academic failure, psychosocial, and behavior problems as disapproval of parents, teachers, and peers regarding the school performance have an impact on the children’s feelings of inferiority and helplessness (Greenham, 1999). These risk factors of psychosocial and behavioral problems among children with reading problems are developmentally cumulative across grades starting early at preschool and primary school (Halonen et al., 2006; Giovagnoli et al., 2020). The complex contextual interaction between various individual, psychosocial, and environmental factors derive a host of internalizing and externalizing mental health problems such as depressive symptoms and disorders (Livingston et al., 2018). Internalizing problems such as anxiety and depression continues throughout childhood and adulthood partly because academic and language skills are important throughout the lifespan (Klassen et al., 2011). Externalizing problem behavior is a result of negative emotions directed against others such as anger, aggression, frustration, conduct disorders, aggressiveness, and antisocial behavior or attention deficit and hyperactivity (Halonen et al., 2006). Long-term, individuals with reading and learning difficulties experience employability issues and lower job satisfaction. Fundamentally, these issues pose a burden at the individual, family, societal, and national level in earnings, increased health expenses, and suicide prevention efforts (Livingston et al., 2018).

Learning to read can be particularly challenging for children from minority ethnic groups or where their first language is not the language of instruction. A growing number of studies on diverse orthographies have highlighted the fact that reading problems occur in all written languages (Borleffs et al., 2019). The prevalence rate of children who exhibit the characteristics associated with specific reading disability (dyslexia) around the world in different orthographies ranges between 5 and 17.5% (Borleffs et al., 2019). It is a particular problem in opaque or less transparent orthographies such as English in comparison to transparent orthographies such as German or Italian (Vellutino et al., 2004). The characteristics that are particularly associated with reading disabilities in alphabetic orthographies include deficiencies in word identification and phonological skills. Reading disability in transparent alphabetic orthographies has also been reported to be related to a reading speed deficit (Landerl, 2001; Vellutino et al., 2004; Wimmer and Schurz, 2010).

Malaysia is a multicultural society comprising three major ethnic groups, Malay, Chinese, and Indian as well as many diverse indigenous cultural groups. The Malaysian states on the island of Borneo, for example, the state of Sarawak is home to 27 ethnic groups with Iban as the largest indigenous group (Department of Information Malaysia, 2016). In Malaysia, children from these diverse ethnic and linguistic backgrounds are expected to be proficient in the Malay language, which is the national language and the medium of instruction in the public education system. In addition, English, being the lingua franca, is the second official language and international language of communication (Ministry of Education Malaysia, 2013). Therefore, every child is expected to achieve bilingual proficiency in both Malay and English language by the time they finish secondary school education (Ministry of Education Malaysia, 2013). This can be particularly challenging for children from minority ethnic groups whose first language is different from the two official languages, Malay and English. In addition, socio-economic status remains as the greatest predictor of academic performance among Malaysian children (Ministry of Education Malaysia, 2013).

At present, there is no comprehensive reading assessment battery that can be used for identifying reading problems in multilingual Malaysia. Only one previous study has assessed the validity of a dyslexia assessment battery in a relatively small sample of Malay children (Lee, 2008). The focus on only one ethnic group limits the applicability to a broader Malaysian multi-ethnic cohort. In addition, word reading fluency and vocabulary measures, which play an important role in transparent orthographies, were not included in that study (Ziegler et al., 2010; Torppa et al., 2016).

The development of a comprehensive reading assessment battery for an early diagnosis of reading difficulties in multilingual children in Malaysian classrooms is of high priority. Thus, the primary objective of the current study was to develop a comprehensive reading battery to aid in the assessment of reading and reading difficulties among children in the Malay language. The second objective was to determine the reliability and validity of this reading assessment battery. The third objective was to analyze the constructs that best account for reading ability, and are thus, useful for identifying children at-risk of reading difficulties. The final objective was to compare results from the reading assessment battery with those from the Malaysian national screening instrument (LINUS; Kang, 2012; Ministry of Education Malaysia, 2018)^1^. The Malaysian national screening instrument (LINUS) is used to screen children and identify children who are at-risk and not-at-risk for later reading difficulties. It includes letter and syllable matching, word reading, and short passage comprehension tests (Kang, 2012; Ministry of Education Malaysia, 2018). The screening tool is administered by classroom teachers during the school year.

### The Malay Language and Classroom Instruction

The Malay language is a member of the Malayo-Polynesian language group (Collins and Ahmad, 1999). It has a highly transparent alphabetic orthography with near perfect and consistent grapheme-phoneme correspondences (Borleffs et al., 2019). It has 26 letters (a–z) similar to the English alphabet. There are three types of sounds in the Malay language: vowels (a, e, i, o, u) with e having 2 vowel sounds such as /e/ for *ekor* (tail) and /ǝ/ for *emak* (mother); diphthongs (ai, au, oi); and consonant sounds (Lee and Wheldall, 2011). There are five digraphs: /gh/, /kh/, /ng/, /ny/, and /sy/. The syllable structures of the Malay language include vowel, vowel consonant, consonant vowel, and consonant vowel consonant (Lee, 2008). It is also an agglutinative language, where affixation (*berjalan*, ‘walking,’ is derived from the base word *jalan*); compounding (*ibu*, ‘mother’ and *bapa*, ‘father’ becomes *ibubapa*, ‘parents’) and reduplication (gopoh becomes gopoh-gapah, ‘haste’) are common characteristics (Rickard Liow and Lee, 2004). The typical teaching of reading approach in Malaysian classrooms is based on the letter-name approach, and which is then blended at the level of syllables and morphemes (Rickard Liow and Lee, 2004). For example, to learn the word *bapa* ‘father,’ the letter names of the first syllable *ba* is spelled and then blended to form /ba/. Then the second syllable in the word *pa* is spelled out and then blended to form /pa/. Finally, the first and second syllable (/ba/ and /pa/ are blended to form the word *bapa*). There is no explicit instruction of grapheme-phoneme correspondences (Winskel and Lee, 2014).

### Multicomponential Approach for Assessing Reading and Reading Disabilities

Language and literacy skills that are important for becoming literate include word recognition, comprehension, vocabulary, fluency, and spelling (National Reading Panel, 2000; Snow, 2006). Reading disabilities are a complex multicomponential deficit, which involves not only phonology but also difficulties with grammar and vocabulary (Tunmer and Greaney, 2010; International Dyslexia Association, 2020). In a recent study, Nation (2019) argued that reading disabilities, captured across time, involves a multifaceted aspect of reading going beyond decoding to include linguistic comprehension including vocabulary and oral language comprehension.

Word reading accuracy has been found to be closely linked to spelling development (Ehri, 1998; Tunmer and Chapman, 2012), as both processes require grapheme-phoneme connections. According to Ritchey (2008), letter name, letter sound, and phonological knowledge foster children’s reading and spelling development. Young children use their alphabetic knowledge to make connections between letters and sounds to decode words. Research on transparent orthographies such as Dutch has shown that both word reading and spelling are related (Schaars et al., 2017). In another study on another relatively transparent orthography, namely German, it was found that improvement in spelling among primary school children was associated with good performance in reading (Moll et al., 2019).

Word reading fluency is an important construct for differentiating between students who struggle with reading and those who do not (Speece and Ritchey, 2005). Moreover, reading fluency at the word, sentence, and passage levels have been reported to be interrelated (Klauda and Guthrie, 2008). Oral reading fluency is an important indicator of reading proficiency (Fuchs et al., 2001) because faster word recognition frees up the cognitive resources for making inferences and using background knowledge in comprehending texts. The more a student comprehends, the more fluent his/her reading is and vice versa, which suggests that a reciprocal relation exists between reading fluency and reading comprehension (Klauda and Guthrie, 2008; see also Little et al., 2017). A recent study on Finnish language, a highly transparent orthography, reported that reading fluency and reading comprehension have a strong correlation in Grade 1 (*r* = 0.72; Torppa et al., 2016). In another study on Spanish, which also features consistent orthography-phonology mappings, it was found that a key feature of reading development is word reading fluency (Davies et al., 2007). The inclusion of a word reading fluency assessment is important in transparent orthographies, as reading accuracy reaches ceiling quickly (Ziegler et al., 2010; Torppa et al., 2016).

Vocabulary, a form of oral language, is an important correlate of reading achievement (Catts et al., 1999; Bowey, 2007; Torppa et al., 2016). Oral language skills contribute to both word-level reading and reading comprehension (Foorman and Connor, 2011). Various studies have found a strong continuity between the inside-out (i.e., code-related) and outside-in (i.e., language-related) skills across Grades 1–3 (Whitehurst and Lonigan, 1998; NICHD Early Child Care Research Network, 2005). As suggested by Nation (2019), vocabulary knowledge can differentiate between poor and good readers.

Listening comprehension refers to the ability to comprehend spoken language verbalized in utterances (Kim and Pilcher, 2016). We draw from studies on English, an opaque orthography, where listening comprehension and oral language skills have been more widely studied. One study by the Language and Reading Research Consortium [LARRC] (2017) reported that listening comprehension and oral language (e.g., vocabulary) in the English language loaded on different factors, yet was highly correlated, and therefore, best defined as one construct in the early elementary school years. In another study examining first graders’ English language early reading skills using exploratory analysis, Kendeou et al. (2009) found two distinct contributing factors: decoding skills where the vocabulary composite loaded together with non-word fluency, oral reading fluency, and retell fluency as one factor while the comprehension skills factor was comprised of listening comprehension and retell fluency. Similarly, Tunmer and Chapman (2012) also found two factors comprising decoding (i.e., word recognition, letter-sound knowledge, and reading comprehension) and linguistic comprehension (listening comprehension and vocabulary knowledge) in first grade children in New Zealand. Turning to transparent orthographies, for example, in the Malay language, Lee (2008) found that listening comprehension loaded with reading comprehension (there was no vocabulary measure in this study). Both listening and reading comprehension measures in Lee’s study could have tapped the same oral language skills given that both questions and answers were administered and answered orally. In terms of the relation between listening comprehension and word reading fluency measures, studies on highly transparent orthographies have reported weak correlations between these two measures (e.g., Finnish, *r* = 0.05, Torppa et al., 2016) but moderate in other highly transparent orthographies (e.g., Malay, *r* = 0.42; Lee, 2008). Thus, inconsistencies have been found in relation to the link between listening comprehension and other reading-related measures.

### Sublexical-Reading and Cognitive Constructs That Characterize Reading Disabilities

Children struggling with learning to read may lack crucial sublexical-reading and cognitive skills (e.g., Al Otaiba and Fuchs, 2002; National Early Literacy Panel [NELP], 2008; Fletcher et al., 2011). These important sublexical-reading and cognitive skills are as follows:

#### Alphabet Knowledge

Early alphabet knowledge of letter names and letter sounds has been found to moderately predict later decoding (*r* = 0.50), reading comprehension (*r* = 0.48), and later spelling skills (*r* = 0.54; National Early Literacy Panel [NELP], 2008) in the English language. The fluency of both letter names and letter sounds are important in ensuring successful reading development (O’Connor and Jenkins, 1999). Letter name fluency optimally predicts reading outcomes at the end of kindergarten and the beginning of first grade (Catts et al., 2009), while letter name knowledge has been found to lose its predictive power by the end of kindergarten (Schatschneider et al., 2004). Research on various alphabetic orthographies demonstrates that alphabet knowledge predicts early reading and spelling (Caravolas et al., 2012) and reading comprehension (Furnes and Samuelsson, 2010; Torppa et al., 2016). There is support from research that letter name knowledge plays a crucial role in learning to read and spell in the Malay language (Winskel and Lee, 2014).

#### Phonological Awareness

Phonological awareness (PA) involves the metacognitive understanding and manipulation of speech sounds at the word-, syllable-, and phoneme-levels (Blachman, 2000). It is well established that PA is the building block for word reading accuracy and other literacy skills such as reading automaticity, reading comprehension, spelling, and writing in alphabetic orthographies (e.g., Wagner et al., 1997; Boscardin et al., 2008; Tunmer and Chapman, 2012). Children’s difficulties in PA affect word decoding and spelling (e.g., Vellutino et al., 2004). Thus, PA plays a foundational role in literacy skill attainment among skilled and unskilled readers (Tunmer and Greaney, 2010; Tunmer and Chapman, 2012). Furthermore, PA has been reported to be a key component in reading development across a range of transparent orthographies such as Finnish, Norwegian, Swedish, and Dutch (Ziegler et al., 2010; Torppa et al., 2012; Verhoeven and Keuning, 2018).

#### Rapid Automatized Naming

Rapid automatized naming (RAN) refers to the speed of naming a series of objects, colors, letters, and digits (Wolf et al., 1986). RAN has been found to uniquely predict both concurrent and future reading-related outcomes (e.g., Compton, 2003; Bowey, 2007) and spelling (Savage et al., 2008). It is particularly predictive of children who are having reading difficulties in opaque orthographies such as English (e.g., Bowey, 2007; Boscardin et al., 2008; Savage et al., 2008). RAN deficits have also been found to be prevalent in transparent orthographies such as Brazilian Portuguese (Germano et al., 2017), Dutch (de Jong and van der Leij, 2003; Verhoeven and Keuning, 2018), German (Landerl and Wimmer, 2008), Finnish (Torppa et al., 2012), Norwegian/Swedish (Furnes and Samuelsson, 2010), and Spanish (Escribano, 2007). In older children, fluency deficits are reported to be much more persistent than phonological deficits in transparent orthographies (Ziegler et al., 2003).

The predictive power of RAN depends on whether the alphanumeric (digits or letters) or non-alphanumeric (objects or colors) measures are employed (Compton, 2003; Schatschneider et al., 2004; Bowey, 2007). In English, RAN digits uniquely predicted decoding skills in first graders but not RAN colors (Compton, 2003). In transparent orthographies, RAN letters and digits have also been found to significantly differentiate readers with and without reading disabilities (de Jong and van der Leij, 2003; Furnes and Samuelsson, 2010; Torppa et al., 2012; Moll et al., 2019).

#### Phonological Memory

Phonological memory (PM) refers to the ability to store phonological information in short-term memory (Wagner et al., 1997; National Early Literacy Panel [NELP], 2008). Phonological memory is important for enabling the reader to channel cognitive resources maximally for decoding and reading comprehension (Wagner et al., 1997) while deficits in phonological memory hamper these reading processes (Al Otaiba and Fuchs, 2002; Swanson et al., 2009). The correlation between PM and reading outcome has been found to be weaker than the correlation between phonological awareness and reading outcome (Wagner et al., 1997; National Early Literacy Panel [NELP], 2008). Wagner et al. (1997) found that PM did not uniquely influence word reading above and beyond PA and RAN skills. However, research has demonstrated that there are differences in reading ability related to PM (Al Otaiba and Fuchs, 2002; Swanson et al., 2009; Hardy et al., 2019). PM taps short-term memory and has been found to be strongly associated with vocabulary knowledge across the life span (Gathercole et al., 1999). In transparent orthographies, PM is associated with reading development (e.g., Dutch: Verhoeven and Keuning, 2018; Finnish: Dufva et al., 2001) while vocabulary knowledge influences reading development (Ziegler et al., 2010).

## Materials and Methods

### Assessment Development

The process of developing the reading assessment battery involved several stages: item development, content validation, and a pilot study. Established measures in the English language commonly used for identifying reading disabilities were reviewed and used as a reference during the development of items and tests (e.g., Good et al., 2001). The Malaysian Grade 1 textbooks and the content/scope and sequence standards were also reviewed (Ministry of Education Malaysia, 2010, 2011). The Malay language textbooks for Grade 1 children reflects the distinct characteristics of Malay orthography. Multisyllabic words that include affixation, compounding, and reduplication are commonly used even for young children in Grade 1. These words include multisyllabic nouns (*lelaki*, ‘man’; Abdul Malek et al., 2012, p. 58); affixation (*mencuci*, ‘washing,’ is derived from the base word *cuci*, ‘wash,’ Abdul Malek et al., 2012, p. 59), compounding (*ibu*, ‘ mother’ and *bapa*, ‘father’ becomes *ibubapa*, ‘parents’); and reduplication (*kawan–kawan*, ‘friends,’ Abdul Malek et al., 2012, p. 69).

Content validity, which examines whether the test measures reflect the appropriate content (Salvia et al., 2007), was determined by an expert panel comprising 14 trained and experienced remedial and language teachers from six schools in Kuching, the capital city of Sarawak. After the expert panel had received a briefing on the objective of the reading assessment and were trained to conduct the tests, they administered the test to a total of 200 children for the pilot study. The mean age of the students was 7.7 years old. Thereafter, the first and fourth authors obtained feedback about the content, duration, format, and delivery of each assessment. Most of the items were judged to be appropriate by the expert panel. The suggestions from the expert panel included reducing the number of words for the spelling, word reading accuracy, and reading comprehension assessments.

### Measures

All the assessments were administered individually except listening comprehension and spelling, which were group administered. The font type for the reading assessment was Comic Sans MS. For all timed measures (i.e., Letter Name Fluency, RAN Digits, Word Reading Efficiency, and Oral Reading Fluency), a stop watch was used to measure durations. The following measures were included in the reading assessment.

#### Reading Comprehension

Reading comprehension was assessed using the same passage as the Oral Reading Fluency (Form A). By design, reading comprehension was assessed only after the oral reading fluency was tested. There were five questions for the Reading Comprehension assessment. The children read the text and then completed the blank spaces with a one- or two-word responses (Keenan and Meenan, 2012). An example of a question in Malay is: *Why was Uncle Karim sad?* Below this question the answer with a blank space: *Uncle Karim was feeling sad because his rabbit* ________ (correct answer: ran away). A correct answer was awarded 1 point, while an incorrect answer was awarded 0 points; the range for the reading comprehension score was 0–5.

#### Spelling

The Spelling assessment assessed the children’s ability to spell the words verbalized by the examiner. Reversals that did not form different letters, except b and d, were accepted. Examples of the spelling items are *susu* (milk) and *Isnin* (Monday). There were 10 words in the spelling list. Spelling was assessed before word reading accuracy as the same 10 words were used for both measures. Scores ranged from 0 to 10.

#### Listening Comprehension

Two pre-recorded dialogs (21 s each) were administered. There were 3 questions for each dialog. After listening to each dialog twice, the students answered the questions on the answer sheet. One dialog was: *I have an announcement to make. This year*, *there are three students who have been selected to represent our school in the sports competition in Kuala Lumpur*. An example of a question from the dialog in Malay was: *How many students have been selected to represent the school?* Then the test administrator continued to read the options aloud: “*A: 5 students*, *B: 3 students*, *C: 2 students*, *D: 1 student*.” The students were required to circle their answer on the answer sheet. The range of scores was 0–6.

#### Letter Name Knowledge

Alphabet knowledge was assessed in two formats, lowercase and uppercase letters. For each format, 26 letters of the alphabet were presented in a random order, displayed in an array of 2 rows with 13 letters per row. The test was scored based on the total number of letters named correctly. Scores ranged from 0 to 26.

#### Letter Name Fluency

All 26 letters in lowercase were randomly arranged in an array of 6 letters per row by 11 rows (i.e., a total of 66 letters). The students were instructed to name the letters as fast as they could from the top row to the next row until all the letters had been named. The total score was the number of correct letters named in 30 s excluding the errors (range between 0–66).

#### Rapid Automatized Naming (RAN)

Four rows comprising 5 numbers (i.e., 1–5) were presented randomly to assess RAN digits. Practice items (i.e., 3, 1, 4, 2, and 5) were introduced to each student before the RAN digit test was administered. The test was discontinued if a student could not name all the digits during the practice session despite the provision of error correction. The total time taken to read all the digits was recorded. Then, the results were converted into digits-per-second scores. The range of scores was 0.16–3.03.

#### Word Reading Accuracy (WRA)

The WRA test is an untimed measure that assesses the student’s ability to read 10 words. These words also appeared in the spelling list. Scores ranged from 0 to 10.

#### Word Reading Efficiency (WRE)

The WRE test is a timed measure that assesses the student’s ability to read as many words as possible from a word list in 30 s. The list was comprised of 60 real words. The range was between 0–57 for Form A and 0–60 for Form B.

#### Oral Reading Fluency (ORF)

The ORF measures the accuracy and rate of reading connected text within 30 s. Any word that is omitted, substituted, or when pauses exceed three seconds is counted as an error. The total score is the total number of words read correctly in 30 s. The children were asked to read aloud two passages. The range was between 0–85 words for Form A and 0–75 words for Form B.

#### Expressive Vocabulary

Expressive vocabulary was measured using the same pictures used for the Receptive Vocabulary test. The students were given the following instructions in Malay while pointing to the first picture: here are some pictures. Provide the name of each picture starting from here. A one-word answer was expected. There were 20 items. The range of scores was 1–20.

#### Receptive Vocabulary

Receptive vocabulary was measured using pictures that were administered orally by the test administrator. By design, expressive vocabulary was assessed before receptive vocabulary. The students were given the following instructions in Malay. For example, here are some pictures. Point to cake. Turn the page, point to butterfly. The students responded by pointing to the picture of his/her choice. The range of scores was 10–20.

#### Elision

Phonological awareness was assessed using the Elision subtest, which measures students’ ability to delete syllables or phoneme(s) from orally presented words. For example, the students were required to say lampu, ‘lamp’ without /lam/. The items were arranged from the easiest to the most difficult (i.e., from the deletion of syllables to the deletion of phonemes). There were 16 items. The range for the Elision measure was 0–16.

#### Phonological Memory

Phonological memory was measured using the digit span test. The student was instructed to listen carefully to the digits read aloud by the test administrator (e.g., 9 4, 8 3, 2 7, 7 3 9, 9 2 5) and to repeat the numbers orally in the correct sequence. The items ranged from 2-digit span to 8-digit span. There were 18 items. The range of scores was 0–16.

### Participants

The participants (47% females, 53% males) were 866 Grade 1 students from 11 randomly selected government primary schools in Kuching, Sarawak, a Malaysian state located on the island of Borneo. The age of the participants was from 6.61 to 7.82 (*M* = 7.13, *SD* = 0.29). The major ethnic groups comprised of the following: Malay (67.1%), Iban (13.9%), Bidayuh (8.3%), Chinese (3.1%), and others (5.9%), which included the indigenous peoples of Sarawak including the Kayan, Kelabit, Melanau, Murut, and Penan. Missing data was 1.7%.

Using the cross-tabulation approach in SPSS, we report the use of the various languages by the participants as their first (mother tongue), second, and third language. For most of the participants, their first language (L1; mother tongue) was Sarawak Malay (B*ahasa Sarawak*) (*n* = 563). The majority of the participants, who reported using Sarawak Malay as their first language, were the Malay children (86.5% of the Malay participants). It is noteworthy that the first language of the other participants included the following languages: Iban (*n* = 119), Malay language (*n* = 69), Bidayuh (*n* = 51), and Chinese (*n* = 18).

In terms of the second language, a majority of the participants spoke the Malay language (*n* = 748) and the majority of the participants who spoke the Malay language were the Malays (89.15% of the Malays). The English language was the next most widely spoken language among the participants albeit only a small number (*n* = 81) followed by Sarawak Malay (*n* = 15). The most widely spoken third language among the participants was English (*n* = 745). This was followed by only two other reported languages, namely the Malay language (*n* = 34) and Sarawak Malay (*n* = 15). Thus, it was apparent from the present study that although the participants were from multi-ethnic groups, the unifying language was Sarawak Malay, a dialect spoken by most of the locals (The Borneo Post, 2010).

### Procedure

Twenty-two undergraduate university students were trained as test administrators. They all attended a 15-h training session. The trained test administrators tested each child individually in a room at the school. The reading assessment lasted between 45 and 80 min depending on the child’s ability. The reading assessment commenced during the second half of the school year of Grade 1 (i.e., August) to ensure that the students had received at least 6 months of formal instruction. Testing was completed within 2 weeks. The test booklets were scored by the test administrators.

## Data Analysis

### Reliability Analysis

Three forms of reliability, namely, alternate-form reliability, test–retest reliability, and interrater reliability were examined. First, the alternate-form reliability was conducted to determine the content sampling reliability of the timed measures. All children in the study were administered an alternate test for all the timed measures within a span of 1 week. The alternate-form reliability indices were derived using Pearson correlations. Guidelines on *r* interpretations were based on Cohen(1988, cited in Pallant, 2016). Second, the test–retest reliability was conducted to ascertain the consistency of the measures across time. During the retest session, which was 1 week after the initial test, approximately, 5% of the participants (*n* = 46) were randomly tested. The consistency of scores between the first and second testing was measured using Pearson correlation coefficients. Third, interrater reliability was conducted on all the measures (approximately 5.5% of the data). For the measures with continuous data, interclass correlation (ICC) was used to calculate the interrater reliability, except the spelling data, which was calculated using Kuder Richardson Formula 20 (KR-20) (Stevens et al., 2004).

### Concurrent Validity: A Comparison of the Reading Assessment Battery With the Malaysian National Screening Test

To determine the concurrent validity of the measures, we computed the correlations between the reading assessment battery measures in the present study and the raw scores from the Malaysian national screening test (LINUS) that were administered by the schools.

### Exploratory Factor Analysis

The dimensionality of the multiple constructs of the reading assessment battery was determined using exploratory factor analysis (EFA) using SPSS 23. In the first step of the EFA, principal axis factoring (PAF) was selected as the method of extracting factors on oblique rotation with Eigenvalues greater than 1 (Brown, 2006). Kaiser–Meyer–Olkin (KMO) and Bartlett’s Test of Sphericity were computed to examine whether the obtained factors were appropriate to examine the relationship among variables. KMO should be greater than 0.6 for appropriate uses of EFA while Bartlett’s statistic should be statistically significant on chi-square distribution. In the second step, to load each item on only one latent factor, the items with communality (*h*^2^) of less than 0.2 or loaded greater than 0.4 on multiple factors or any item that does not have a factor loading of at least 0.3 on any factor was dropped (DeVellis, 1991; Spector, 1992; Hatcher, 1994). After removing the items above, EFA was re-conducted where factor loadings with an absolute value less than 0.32 were ignored (Tabachnick and Fidell, 2014).

### Categorization of Children With Deficits in Reading

The cut-off-point of 25th percentile (based on the overall mean) was used to determine the presence of deficits across all measures within each of the three factors. This method of using a percentile cut-off-point has been used in numerous studies (e.g., Snellings et al., 2009; Germano et al., 2017).

### Discriminant Analysis of At-Risk and Non-at-Risk Children

Discriminant analysis, a statistical method for classifying the dependent variables between two or more categories, was used to determine which continuous variables discriminate between the two groups of children included in the Malaysian national screening test (LINUS) (i.e., children who were at-risk and not-at-risk for reading difficulties). LINUS is a national literacy screening instrument that includes letter and syllable matching, word reading, and short passage comprehension tests (Kang, 2012; Ministry of Education Malaysia, 2018). The screening tool is administered by teachers during the school year. The LINUS results in the present study were from the second screening session that occurred in July. The Year 1 children were screened for any signs of reading difficulties in the Malay language and were categorized into either at-risk (for children who did not master the screening test) or not-at-risk (for children who mastered the constructs in the screening test). Children who are not able to master the 12 constructs are considered to be at-risk for reading difficulties. Children who are at-risk for reading difficulties based on the screening procedure would then be provided with remediation sessions by the Malay language remedial teacher. For the purposes of this discriminant analysis, children who were classified as at-risk for reading difficulties were coded as “1” and the children who were not-at-risk for reading difficulties were coded as “2” in SPSS. The composite scores of the three factors (Phonological-Decoding, Sublexical-Fluency, and Vocabulary-Memory) for testing the significance of a set of discriminant functions were computed.

## Results

### Descriptive Statistics

Descriptive statistics and correlation analyses are presented in Tables 1, 2, respectively. There was a near ceiling effect for letter name knowledge (capital letters, *M* = 24.82, *SD* = 3.70; small letters, *M* = 24.83, *SD* = 3.70) where 79.3% of the participants scored full points for capital letters and 75.5% of the participants scored full points for small letters. However, a small percentage of about 3% participants were not able to name the letters. Receptive vocabulary also had a near ceiling effect where 75.2% of the participants scored full points. In contrast, there were fewer participants who scored full points for Expressive Vocabulary (*n* = 348, 40.2%). All the variables were significantly correlated at an alpha level of 0.01. The reading measures such as Reading Comprehension, Spelling, Elision, Word Reading Efficiency, and Oral Reading Fluency had a correlation coefficient ranging from 0.66 to 0.86. The Listening Comprehension measure was also significantly correlated with the vocabulary measures. The timed reading measures were significantly correlated (*r* = 0.96). Several correlation coefficients were larger than 0.92. These were Word Reading Efficiency Form A and B, Oral Reading Fluency Form A and B, Letter Name Knowledge (capital letters) and Letter Name Knowledge (small letters), and Letter Name Fluency Form A and B. Large correlation coefficients indicates multicollinearity between two or more variables. To solve the issue of multicollinearity, four measures [Word Reading Efficiency Form A, Oral Reading Fluency Form A, Letter Name Knowledge (Capital Letters), and Letter Name Fluency Form A] were excluded from the final exploratory factor analysis (see also Exploratory Factor Analysis).

**TABLE 1:** Descriptive statistics of total dataset (*N* = 866).

Variable	Mean	(*SD*)	Minimum – Maximum
Reading comprehension^a^	1.96	(1.83)	0.00 – 5.00
Spelling^a^	4.84	(3.23)	0.00 – 10.00
Listening comprehension^a^	3.21	(1.86)	0.00 – 6.00
Letter name knowledge (capital letters)^b^	24.82	(3.70)	0.00 – 26.00
Letter name knowledge (small letters)^b^	24.83	(3.70)	0.00 – 26.00
Letter name fluency Form A^b^	33.66	(12.91)	0.00 – 66.00
Letter name fluency Form B^b^	34.58	(13.25)	0.00 – 66.00
Rapid automatized naming Form A (digits per second)^c^	1.38	(0.47)	0.16 – 3.03
Rapid automatized naming Form B (digits per second)^d^	1.36	(0.46)	0.16 – 2.94
Word reading accuracy^b^	7.34	(3.61)	0.00 – 10.00
Word reading efficiency Form A^b^	16.66	(14.40)	0.00 – 57.00
Word reading efficiency Form B^b^	15.36	(15.05)	0.00 – 60.00
Oral reading fluency Form A^b^	21.69	(20.93)	0.00 – 85.00
Oral reading fluency Form B^b^	22.67	(19.56)	0.00 – 75.00
Expressive vocabulary^b^	18.42	(2.37)	1.00 – 20.00
Receptive vocabulary^b^	19.66	(0.80)	10.00 – 20.00
Elision^b^	9.40	(4.98)	0.00 – 16.00
Phonological memory^b^	9.34	(2.18)	0.00 – 16.00

**As a result of missing data, ^*a*^n = 863; ^*b*^n = 865; ^*c*^n = 860; ^*d*^n = 859.**

**TABLE 2:** Intercorrelations between the assessment measures.

Measure	1	2	3	4	5	6	7	8	9	10	11	12	13	14	15	16	17	18
(1) Reading comprehension	1																	
(2) Spelling	0.80	1																
(3) Listening comprehension	0.58	0.54	1															
(4) Letter name knowledge^a^	0.30	0.42	0.26	1														
(5) Letter name knowledge^b^	0.29	0.40	0.26	0.96	1													
(6) Letter name fluency Form A	0.58	0.67	0.45	0.58	0.57	1												
(7) Letter name fluency Form B	0.57	0.67	0.44	0.59	0.58	0.92	1											
(8) Rapid automatized naming Form A	0.48	0.53	0.38	0.35	0.34	0.71	0.72	1										
(9) Rapid automatized naming Form B	0.46	0.50	0.37	0.35	0.34	0.71	0.71	0.85	1									
(10) Word reading accuracy	0.66	0.80	0.45	0.57	0.54	0.73	0.74	0.53	0.50	1								
(11) Word reading efficiency Form A	0.81	0.85	0.56	0.35	0.33	0.71	0.70	0.60	0.59	0.73	1							
(12) Word reading efficiency Form B	0.79	0.81	0.55	0.31	0.29	0.67	0.67	0.57	0.57	0.67	0.97	1						
(13) Oral reading fluency Form A	0.81	0.83	0.56	0.32	0.30	0.68	0.68	0.58	0.57	0.69	0.96	0.96	1					
(14) Oral reading fluency Form B	0.82	0.86	0.55	0.35	0.33	0.72	0.72	0.59	0.59	0.75	0.96	0.95	0.97	1				
(15) Expressive vocabulary	0.32	0.36	0.29	0.35	0.35	0.33	0.35	0.30	0.28	0.38	0.33	0.32	0.32	0.34	1			
(16) Receptive vocabulary	0.24	0.26	0.21	0.34	0.36	0.27	0.28	0.20	0.17	0.30	0.25	0.24	0.24	0.25	0.39	1		
(17) Elision	0.66	0.72	0.48	0.40	0.39	0.58	0.58	0.44	0.45	0.69	0.71	0.69	0.70	0.71	0.33	0.24	1	
(18) Phonological memory	0.32	0.36	0.32	0.27	0.26	0.33	0.32	0.29	0.29	0.38	0.35	0.33	0.33	0.34	0.34	0.23	0.36	1

### Reliability and Validity Analysis

Three forms of reliability, namely, alternate-form reliability, test–retest reliability, and interrater reliability were examined.

#### Alternate-Form Reliability

For content sampling reliability of the timed measures (i.e., Letter Name Fluency, Rapid Automatized Naming, Word Reading Efficiency, and Oral Reading Fluency), there were small differences between the alternate forms in terms of the means and standard deviations for each timed measure. The alternate-form reliability indices of these timed measures were high. For Word Reading Efficiency, Oral Reading Fluency, Letter Name Fluency and Rapid Automatized Naming, *r* was above 0.85 (range: 0.85 to 0.97; see Table 2).

#### Test–Retest Reliability

Time sampling was performed using the test–retest method to investigate whether a student’s test performance is constant over time. Table 3 shows that *r* was between 0.72 and 0.98 suggesting that there was acceptable to excellent coefficient stability over time, except for the Phonological Memory measure which had a lower coefficient than the rest of the measures (*r* = 0.69).

**TABLE 3:** Test–retest reliability of untimed measures.

Measures	First testing^a^		Second testing^b^		*r*
	Mean	*SD*	Mean	*SD*	
Letter name knowledge (capital letters)	24.83	3.72	25.63	1.83	0.83
Letter name knowledge (small letters)	24.83	3.70	25.80	0.54	0.72
Word reading accuracy	7.34	3.61	8.67	2.80	0.98
Expressive vocabulary	18.42	2.37	19.23	1.36	0.79
Receptive vocabulary	19.66	0.80	19.91	0.28	0.73
Elision	9.43	4.98	11.93	4.27	0.74
Phonological memory	9.34	2.18	9.93	2.07	0.69

**^*a*^N = 865; ^*b*^N = 46; SD, standard deviation; r, correlation coefficient*.*

#### Interrater Reliability

The interclass correlation (ICC) on 5.5% of the participants per measure exceeded 0.9. For the binary judgment of spelling outcomes, we used the Kuder Richardson Formula 20 (KR-20) (Stevens et al., 2004), which yielded coefficients that exceeded 0.9 between the interraters for all spelling items on the entire data set.

### Concurrent Validity: A Comparison of the Reading Assessment Battery With the Malaysian National Screening Test

All the correlation coefficients between the raw scores from the Malaysian national screening test (LINUS) and the measures in the present study were significant. The highest correlations were between the LINUS screening test and Word Reading Accuracy (*r* = 0.82) and Spelling (*r* = 0.67). This was followed by the alternate forms of Word Reading Efficiency (*r* = 0.59 and 0.53), Oral Reading Fluency (*r* = 0.55 and 0.60), and Reading Comprehension (*r* = 0.53).

### Exploratory Factor Analysis

Based on the selection rule described in the data analysis section, the following five measures among 18 measures were removed: Word Reading Accuracy, Word Reading Efficiency Form A, Oral Reading Fluency Form A, Letter Name Fluency Form A, and Letter Name Knowledge (Capital Letters). After the removing the 5 measures, the EFA with 13 tests (see Table 4) was conducted. Three factors with eigenvalues greater than 1 were extracted. Seventy percent of the total variance in the 13 tests was explained by the extracted three factors. KMO greater than 0.9 and the statistically significant Bartlett’s Test of Sphericity [χ^2^ 426 (78) = 85598, *p* < 0.001] suggested the appropriateness of the obtained factors. All factor loadings were greater than 0.32 (Tabachnick and Fidell, 2014).

**TABLE 4:** Factor loadings and communalities (*h*^2^) from the exploratory factor analysis.

Item	Factor 1	Factor 2	Factor 3	*h*^2^
Reading comprehension	0.93			0.87
Spelling	0.87			0.77
Listening comprehension	0.54			0.31
Elision	0.69			0.50
Word reading efficiency^b^	0.95			0.93
Oral reading fluency^b^	0.96			0.94
Rapid automatized naming^a^		0.93		0.87
Rapid automatized naming^b^		0.98		0.97
Letter name fluency^b^		0.58		0.42
Expressive vocabulary			0.62	0.39
Receptive vocabulary			0.57	0.33
Phonological memory			0.36	0.16
Letter name knowledge^c^			0.42	0.23

**Only loadings above 0.30 and above are displayed. *h*^2^ = communalities of the measured variables. ^*a*^Form A. ^*b*^Form B. ^*c*^Small letters.**

The first factor, which we refer to as Phonological-Decoding, comprised 6 measures (Reading Comprehension, Spelling, Listening Comprehension, Elision, Word Reading Efficiency Form B, and Oral Reading Fluency Form B). The second factor, which we refer to as Sublexical-Fluency, comprised 3 measures (Rapid Automatized Naming Form A, Rapid Automatized Naming Form B, and Letter Name Fluency Form B). The third factor, which we refer to as Vocabulary-Memory, comprised 4 measures (Expressive Vocabulary, Receptive Vocabulary, Phonological Memory, and Letter Name Knowledge Small Letters) (see Table 4).

### Categorization of Children at Risk of Reading Difficulties

We also classified our sample according to the percentile scores. Table 5 shows the at-risk classification and number of students according to the distribution of factors.

**TABLE 5:** Distribution of the factors according to the cut-off at risk criterion below the 25th percentile.

Factors	Assessment	*n* risk criteria < 25th percentile (%)	Scores at 25th percentile	Scores at 75th percentile
Phonological-decoding	Reading comprehension	293 (33.83%)	0	4
	Spelling	269 (31.2%)	2	8
	Listening comprehension	348 (40.3%)	2	5
	Elision	222 (25.7%)	5	14
	Word reading efficiency^b^	227 (26.2%)	2	24
	Oral reading fluency^b^	210 (24.3%)	4	38
Sublexical-fluency	Rapid automatized naming^a^	203 (23.6%)	1.04	1.68
	Rapid automatized naming^b^	210 (24.4%)	1.02	1.66
	Letter name fluency^b^	209 (24.2%)	26	43
Vocabulary-memory	Expressive vocabulary	309 (35.7%)	18	20
	Receptive vocabulary	214 (24.7%)	20	20
	Phonological memory	279 (32.3%)	8	11
	Letter name knowledge^c^	211 (24.4%)	26	26

*^*a*^Form A. ^*b*^Form B. ^*c*^Small letters.*

### Discriminant Analysis

Based on the composite scores of the three factors (Phonological-Decoding, Sublexical-Fluency, and Vocabulary-Memory) for testing the significance of a set of discriminant functions, the results of the discriminant analysis on the testing of significance of a set of discriminant functions (see Table 6) are as follows. The Wilk’s Lambda (*F*-test) showed that the model was a good fit for the data and could be used for the prediction of group membership, Omnibus Wilks’ Λ = 0.521, χ^2^ = 540.788, *p* < 0.01. Examining the discriminant function using the group centroids, the results showed that this discriminant function separates the at-risk group (function = −1.579) from the non-at-risk group (function = 0.582). The standardized canonical discriminant function coefficient for the strongest function, Phonological-Decoding, was 0.573, the coefficient for Sublexical-Fluency, the second strongest function was 0.411, and the coefficient for Vocabulary-Memory was 0.276. In general, all of the three factors were useful in predicting the two groups. Weighing the relative importance of predictors to the discriminant function, Phonological-Decoding was considered to be the best construct that could distinguish between at-risk from non-at-risk children.

**TABLE 6:** Summary of the test of significance of a set of discriminant functions.

Independent variable	Unstandardized	Standardized	Discriminant loading (rank)	Univariate *F* ratio
Phonological decoding	0.017	0.573	0.864 (1)	571.069**
Sublexical fluency	0.038	0.411	0.829 (2)	525.240**
Vocabulary memory	0.054	0.276	0.593 (3)	269.181**
Group centroid low			−1.579	
Group centroid high			0.582	
Wilks Lambda			0.521*	
(Canonical correlation)^2^			0.479	

**p < 0.01. ^2^Squared canonical correlation.

## Discussion

In this study, we developed a comprehensive reading assessment battery in the Malay language (the official language of Malaysia) for children from multi-ethnic and multilingual backgrounds. We also examined the reliability and validity of the reading assessment battery. Subsequently, we also determined the dimensionality of the factors that best explained reading difficulties in the Malaysian language, a highly transparent alphabetic orthography. Finally, we used a national screening instrument in the Malay language to validate the multicomponential reading assessment battery based on children who had been identified as being “at-risk” and “not-at-risk” for reading difficulties using the national screening instrument (LINUS). This is the first comprehensive reading assessment battery that has been developed in Malay for children from multi-cultural and multilingual backgrounds. The participants for the study included a representative sample of children from diverse backgrounds in Malaysia.

### Key Findings

Notably, reading fluency measures such as word reading efficiency and oral reading fluency, which assess the speed of reading of single words and connected text, respectively, were included in the current study. Timed measures have been found to be particularly important in discriminating reading difficulties once word reading accuracy has reached ceiling in transparent orthographies (Ziegler et al., 2010). In the current study, high correlations between reading comprehension and word reading efficiency, a timed word reading test, were found. This corroborates previous findings in other highly transparent orthographies (Torppa et al., 2016). Reading fluency measures have been widely used in studies on early reading achievement and reading disabilities in the English language and transparent orthographies (e.g., Landerl, 2001; Furnes and Samuelsson, 2010; Tunmer and Greaney, 2010; Wimmer and Schurz, 2010; Ziegler et al., 2010; Torppa et al., 2016).

In relation to letter name knowledge, we found a ceiling effect. Children were assessed in the second half of the school year. A plausible explanation is the teaching sequence of letter name knowledge (Schatschneider et al., 2004). In Malaysia, children name the letters and then blend the syllables using the letter names to derive a word. Similar findings have been reported by Torppa et al. (2016) in Finnish children, who also reached a plateau in letter name knowledge toward the end of kindergarten. In that study, the correlation between letter knowledge and other measures such as listening comprehension, reading comprehension, and word reading fluency were weak to moderate.

### Assessment of the Validity and Reliability of the Reading Assessment Battery

High reliability and validity of the measures in the reading assessment battery were found. The overall psychometric features of the reading battery were met in terms of reliability (i.e., alternate-form reliability and test–retest reliability) and concurrent validity. The test of concurrent validity, which was conducted to examine how well the reading assessment battery compared with the LINUS Malaysian national screening test, showed moderate to strong correlations between the reading assessment battery and the national screening test suggesting that both the screening test and the reading battery measure shared constructs. The alternate-form reliability correlation coefficients on the timed tests were high. Results from the time sampling reliability coefficients demonstrated that there was a high measurement consistency for the same respondents at a specified time interval. These results suggest the possibility of scaling up the use of the reading assessment battery to other locations.

### Dimensionality of the Reading Assessment Battery

The exploratory factor analysis conducted resulted in a three-factor solution; one factor which comprised reading measures and related-measures (elision), another factor comprised the sublexical fluency measures including rapid automatized naming and letter name fluency. The third factor was comprised of oral language measures including receptive and expressive vocabulary, letter name knowledge, and phonological memory. In the final EFA model, a 3-factor model, which comprised 13 measures, was extracted. Factor 1 (Phonological-Decoding) was comprised of reading comprehension, spelling, listening comprehension, elision, word reading efficiency, and oral reading fluency. The research findings indicated that phonological-decoding skills including word reading efficiency, oral reading fluency, reading comprehension, spelling, and elision, were strongly correlated. These constructs loaded together on the first factor because the common abilities required to perform word-level reading (i.e., word reading efficiency), passage-level reading (oral reading fluency, reading comprehension), and encoding (i.e., spelling) are phonological awareness, alphabetic principle, and decoding of words and reading connected text. Word reading and reading comprehension have been reported in past studies to load as one factor given that reading comprehension requires the ability to decode (Tunmer and Chapman, 2012; Torppa et al., 2016). Elision loaded together with reading measures and spelling as Factor 1 because there is a close relationship between spelling, reading, and phonological awareness (Ziegler et al., 2010; Tunmer and Chapman, 2012). Elision, which is a subcomponent of phonological awareness, is extremely crucial for word-level reading, passage-level reading, and reading comprehension in various orthographies (Boscardin et al., 2008; Lee, 2008; Lee and Wheldall, 2009; Caravolas et al., 2012). The present study supports previous research that has suggested that phonological awareness is a universal predictor of reading across alphabetic orthographies of varying orthographic depth (Ziegler et al., 2010). Phonological awareness is an important component in reading assessment batteries in an alphabetic transparent orthography such as Malay. Direct reading measures such as reading comprehension, spelling, listening comprehension, word reading efficiency, and oral reading fluency and the sublexical-reading and cognitive constructs such as elision that characterize reading difficulties/disabilities should be included in reading assessment batteries given its utility in transparent orthographies (see Torppa et al., 2016).

In the present study, the word-level and passage-level reading measures loaded as one factor. These findings are similar to other studies on English orthography (Ehri, 1998; Kendeou et al., 2009; Tunmer and Chapman, 2012) where decoding-related skills were highly correlated and/or loaded together as a factor. It is also similar to an earlier study on Malay orthography, where several measures such as non-word reading, passage reading, spelling, elision, word reading, and reading comprehension loaded on the phonological-decoding factor (Lee, 2008). The present findings suggest that there are similarities between alphabetic orthographies in terms of the decoding construct and its corresponding measures. The similarity across these transparent and non-transparent languages is useful for conceptualizing and operationalizing the important constructs when using reading assessment batteries for identifying reading difficulties and disabilities in Malaysia, where both Malay and English are taught in schools concurrently.

It is surprising that listening comprehension was identified as a Phonological-Decoding factor despite the significant but moderate correlation between listening comprehension and the reading-related measures (e.g., Word Reading Accuracy, *r* = 0.45). The current finding diverges from that reported by Tunmer and Chapman (2012), who found that listening comprehension and reading comprehension formed two separate factors. These findings also differ from Kendeou et al. (2009) study, where vocabulary loaded on the decoding skills factor while listening comprehension loaded on the comprehension skills factor together with the retell fluency measure. It is plausible that if the assessments were in the format of a simple oral retell task (Kendeou et al., 2013), listening comprehension might have loaded on another factor. Thus, the present finding has implications for future improvements in the format of the listening comprehension measure.

Sublexical-Fluency, the second factor comprised of rapid automatized naming and letter name fluency, which are needed for reading fluency. Studies on reading in transparent orthographies have found that speed of processing tests such as RAN and letter name fluency appear to characterize children with reading disabilities (de Jong and van der Leij, 2003). Given that RAN and PA loaded as separate factors supports past findings that RAN is not a subset of PA (Norton and Wolf, 2012). The present study supports the importance of including sublexical processing speed measures in reading assessment batteries in transparent orthographies (de Jong and van der Leij, 2003; Torppa et al., 2012; Germano et al., 2017; Verhoeven and Keuning, 2018).

Vocabulary-Memory, the third factor comprised of expressive vocabulary, receptive vocabulary, phonological memory, and letter name knowledge. In the present study, it is plausible that vocabulary, phonological memory, and letter name knowledge loaded together given that these measures are related to memory ability. A deficit in phonological memory is related to reading development and reading difficulties (Dufva et al., 2001; Verhoeven and Keuning, 2018). Gathercole et al. (1999) reported that there is a robust association between phonological memory and vocabulary across the life span for young children to teenagers. Phonological memory and letter name knowledge require children to have the ability to recall from short-term memory. In a study of Brazilian first graders using a factor analysis of a battery of literacy assessments, it was reported that auditory comprehension of sentences from pictures and phonological memory loaded as a factor (Germano et al., 2017). Thus, despite the weaker contribution of vocabulary and memory related measures such as phonological memory toward reading, these are important measures to be assessed.

Altogether, these three factors provide important insights into the constructs that need to be included when assessing young children in relation to reading difficulties/disabilities in the Malay language. Future reading assessment batteries need to focus on phonological-decoding, sublexical-fluency, and vocabulary-memory skills. Focusing on decoding skills alone when assessing young children for reading difficulties/disabilities is insufficient because the speed of processing or retrieval of sublexical information such as letter names and/or digits from long-term memory may be a secondary cognitive deficit that deserves an equal focus by clinicians, teachers, and researchers. Phonological memory, which taps the ability to channel cognitive resources optimally, is necessary in the reading process. In conclusion, the present findings demonstrate that factors that describe reading difficulties/disabilities in an alphabetic orthography such as Malay resembles findings from other alphabetic orthographies regardless of orthographic depth (Ziegler and Goswami, 2005; Borleffs et al., 2019).

### Categorization Criteria for Children at Risk of Reading Difficulties

Based on our categorization criteria of using the 25th percentile as the cut-off point, we found that 24–35% of the first graders were considered to be at-risk of reading difficulties dependent on the reading abilities being assessed. This study highlights the importance of using a comprehensive reading assessment battery. Using a cut-off point of the 10th percentile and 40th percentile on reading, decoding, and spelling, one study on Standard Indonesian reported a prevalence rate of 17.3% at-risk children in Grade 1 (Jap et al., 2017). It is important to note that the children assessed as at-risk in the present study may not actually develop reading disabilities in the future. Given that explicit phonics instruction is not yet implemented in Malaysian classrooms, future studies are necessary to examine the impact of high-quality instruction in relation to fostering reading acquisition and combating reading difficulties. The present study has developed the first comprehensive reading assessment battery for multilingual children in Malaysia. This will be a useful instrument in the early assessment of children’s reading and reading-related skills as well as in the implementation of future literacy intervention programs.

### Discriminating Between at-Risk and Non-at-Risk Children

The diagnostic value of the reading assessment battery was examined and validated against the countrywide Malaysian national screening instrument (LINUS). Discriminant analysis was used to assess the extent to which the assessment battery could reliably discriminate between groups of children who had been identified for being at risk or not at risk for reading difficulties through LINUS. It was found that the reading assessment battery effectively distinguished between the at-risk and not-at-risk children on Phonological-Decoding, Sublexical-Fluency, and Vocabulary Memory with Phonological Decoding being superior for discriminating between the two groups of children. The present study highlights the importance of reading and reading-related measures that are useful for discriminating between children at-risk and not-at-risk for reading difficulties in the multicultural and multilingual Malaysian context.

### Implications for Policy and Practice

The reading assessment battery comprising language, literacy, and sublexical-reading measures has been developed at an important time. The findings bear important implications on the types of measures that can be used to identify children at-risk of developing reading difficulties. This enables appropriate interventions to be provided at an early stage in children’s literacy development.

### Limitations and Future Research

The present study has some limitations, which can be remedied in future studies. First, the study did not include letter-sound fluency and non-word tests, which are important predictors of reading. These assessments were excluded because letter-sound correspondences are not explicitly taught in Malaysian schools and the pilot study results showed that there were floor effects for letter sound knowledge. Similar findings have been reported by Lee and Wheldall (2011), as they found that Grade 1 children were unable to sound out letter sounds in Malay. The present study can be extended to include an assessment of a systematic phonics instruction program that incorporates letter-sound correspondence as well as larger units such as syllables and morphemes (see Ziegler and Goswami, 2005; Lyytinen et al., 2015).

It is important to develop a systematic approach to teaching that includes phonics instruction, spelling, vocabulary, fluency, and reading comprehension (National Reading Panel, 2000). Further studies on the instructional strategies used in classrooms will provide valuable information on reading acquisition in Malaysian schools. Another consideration is that the listening comprehension measure in this study required students to choose one of four options after the answers were read to them. Future research could also include responses that are elicited through retelling and pointing to pictures that describe the story. Third, an increased number of children could be recruited in future studies. Fourth, future research should examine the difficulties encountered in reading multi-syllabic words in Malay, which could pose significant challenges when learning to read Malay. Fifth, a longitudinal study of reading development and monitoring of reading difficulties among children from different socio-economic backgrounds in a wider geographical location is warranted. This would also allow us to examine the proportion of children diagnosed as at-risk and not-at-risk and whether they develop later reading difficulties. Finally, the present study has focused on a narrow academic aspect of reading difficulties. Future research is warranted to focus on the holistic identification of reading difficulties and the evaluation of the ecological, emotional, psychosocial, behavioral aspects, and strategies that are predictive of the overall success of individuals with reading disabilities and learning disabilities (Giovagnoli et al., 2020).

## Data Availability Statement

All datasets presented in this study are included in the article/Supplementary Material.

## Ethics Statement

The studies involving human participants were reviewed and approved by Ministry of Education Malaysia’s Division on Planning and Educational Research. Written informed consent to participate in this study was provided by the participants’ legal guardian/next of kin.

## Author Contributions

The study reported here was realized in cooperation between the authors. JL is the main author of this work. The conception and research design was done by JL. Data collection were organized by JL, PO, and ZN. Statistical analyses were done by JL, SL, and NY. JL made the most contribution toward writing, literature review, and revising the manuscript. SL and JL contributed toward writing up the statistical analyses. HW made substantial contributions by providing feedback and revising the manuscript. All authors contributed to the article and approved the submitted version.

## Conflict of Interest

The authors declare that the research was conducted in the absence of any commercial or financial relationships that could be construed as a potential conflict of interest.

## Footnotes

1 LINUS is a national literacy (English and Malay Language) and numeracy screening tool. The LINUS data in the present study were obtained from the Department of Education Malaysia.
